# Citrullination of fibronectin modulates synovial fibroblast behavior

**DOI:** 10.1186/ar4083

**Published:** 2012-11-05

**Authors:** Miriam A Shelef, David A Bennin, Deane F Mosher, Anna Huttenlocher

**Affiliations:** 1 Department of Medicine, Division of Rheumatology, University of Wisconsin - Madison, 1685 Highland Avenue, Madison, WI 53705, USA; 2 Department of Pediatrics, University of Wisconsin - Madison, 1550 Linden Drive, Madison, WI 53706, USA; 3 Department of Medical Microbiology and Immunology, University of Wisconsin - Madison, 1550 Linden Drive, Madison, WI 53706, USA; 4 Department of Biomolecular Chemistry, University of Wisconsin - Madison, 1300 University Avenue, Madison, WI 53706, USA

## Abstract

**Introduction:**

Rheumatoid arthritis is an autoimmune arthritis characterized by joint destruction. Anti-citrullinated protein antibodies are pathologic in rheumatoid arthritis, but the role of the citrullinated proteins themselves is much less clear. Citrullination is the conversion of the arginine residues of a protein to citrulline. In the inflamed rheumatoid joint there is increased protein citrullination. Several proteins are citrullinated in rheumatoid arthritis, including collagen type II, fibrinogen, and fibronectin. Fibronectin is thought to mediate the adhesion of joint-invading synovial fibroblasts to the rheumatoid cartilage in addition to regulating other synovial fibroblast functions. However, the effect of citrullinated fibronectin on synovial fibroblasts is unknown.

**Methods:**

To investigate the effect of citrullinated fibronectin on synovial fibroblast behavior, we cultured normal murine, arthritic murine, and human rheumatoid synovial fibroblasts. We then compared several synovial fibroblast functions in the presence of fibronectin versus citrullinated fibronectin. We assessed adhesion with time-lapse microscopy, migration with transwell assays, focal adhesion kinase and paxillin phosphorylation by western blot, and focal matrix degradation by fluorescent gelatin degradation.

**Results:**

Normal synovial fibroblasts have impaired adhesion, spreading, migration, and integrin-mediated phosphorylation of focal adhesion kinase and paxillin on citrullinated fibronectin. Murine arthritic and human rheumatoid synovial fibroblasts also have impaired adhesion and spreading on citrullinated fibronectin, but focal matrix degradation is unaffected by citrullinated fibronectin.

**Conclusion:**

Citrullination of fibronectin alters synovial fibroblast behavior and may affect how these cells adhere to and invade the joint and travel through the bloodstream. This work suggests an important role for the interaction of synovial fibroblasts with citrullinated matrix in the pathophysiology of rheumatoid arthritis.

## Introduction

Rheumatoid arthritis is a chronic, debilitating arthritis characterized by painful joint inflammation and destructive erosions. Rheumatoid arthritis has long been known to be an inflammatory arthritis, but only recently has it been shown to be a true autoimmune disease with an immune response generated against self-antigens. The key to this finding was the identification of anti-citrullinated protein antibodies (ACPAs), which contribute to arthritis by forming immune complexes that are deposited in the joint [1] and by activating complement [2]. ACPAs are specific for rheumatoid arthritis and predict severe, erosive arthritis [3].

Despite the rapidly increasing volume of information about ACPAs, the role of the citrullinated proteins themselves is less clear. Citrullination is the conversion of a protein's arginine residues to citrulline, resulting in a loss of charge and often abnormal protein conformation and function. Citrullination is catalyzed by a family of enzymes called peptidyl arginine deiminases (PADs). Protein citrullination in the rheumatoid joint is increased [4] and PAD4 and PAD2 are upregulated in rheumatoid synovium [5,6] and synovial fluid [4]. Inflammation appears to play a role in the level of citrullination since local administration of glucocorticoids reduces citrullination in the rheumatoid joint [7]. Despite the specificity of ACPAs to rheumatoid arthritis, however, citrullination is a more generalized phenomenon - with increased citrullination seen in the synovial fluid of inflamed joints affected by spondyloarthropathy [4] as well as in inflamed muscle in myositis and [8] myelin basic protein in multiple sclerosis [9].

The role of protein citrullination in rheumatoid arthritis is enigmatic, although most evidence supports a pathologic role. Citrullinated fibrinogen [10] and citrullinated collagen type II [11] are more immunogenic and arthritogenic in mouse models of arthritis, and citrullinated fibrinogen activates macrophages more than unmodified fibrinogen [12]. Further, treatment with Cl-amidine, a pan-PAD inhibitor, improves collagen-induced arthritis [13]. In contrast, citrullinated CXCL10, CXCL11 [14], IL-8 [15], and CXCL12 [16] lose inflammatory function, but these proteins have not been shown to be citrullinated in rheumatoid arthritis.

Some proteins that have been shown to be citrullinated in rheumatoid arthritis include type II collagen, vimentin, fibrinogen, and fibronectin [17,18]. Fibronectin is interesting because it modulates numerous cellular behaviors including migration, adhesion, invasion, and survival. More specific to rheumatoid arthritis, fibronectin is deposited on the surface of articular cartilage in the rheumatoid joint [19] and increases the ability of synovial fibroblasts to adhere to cartilage [20].

Synovial fibroblasts are cells that normally line the joint. These fibroblasts play a significant role in rheumatoid arthritis by increasing in number as part of a pannus and by degrading cartilage and bone using matrix metalloproteases and invasive structures called invadopodia [21]. Synovial fibroblasts also can migrate in the bloodstream to invade distant cartilage in mouse models [22], which may explain how multiple joints are involved in rheumatoid arthritis.

Fibronectin plays an important role in synovial fibroblast behavior. Synovial fibroblasts express the integrins α_5_β_1 _and α_v_β_3_, which are important for fibronectin binding [23], and fibronectin is present throughout the pannus [24]. Further, fibronectin mediates synovial fibroblast adhesion to cartilage, stabilizes invadopodia [25], and modulates matrix metalloprotease production [26].

Despite the importance of fibronectin in synovial fibroblast and arthritis pathology, almost nothing is known about the function of citrullinated fibronectin. We know only that there is increased affinity of citrullinated fibronectin for vascular endothelial growth factor but decreased affinity for β_1 _integrin and decreased ability of citrullinated fibronectin to cause apoptosis of HL-60 cells [27].

Given the presence of citrullinated fibronectin in rheumatoid arthritis, the paucity of information about citrullinated fibronectin, and the importance of the interactions of fibronectin and synovial fibroblasts in rheumatoid arthritis, we decided to investigate how citrullinated fibronectin modulates synovial fibroblast behavior. We report in this paper that citrullinated fibronectin leads to impaired adhesion, spreading, migration, and integrin-mediated signaling of synovial fibroblasts, yet focal degradation is unchanged.

## Materials and methods

### Patients

De-identified synovial fluid samples from patients given a diagnosis of rheumatoid arthritis by a rheumatologist were provided by physicians not associated with this research. Joint aspiration was part of routine care for the patients. This research does not meet the definition of human subjects research or require informed consent as confirmed by the University of Wisconsin Institutional Review Board.

### Animals

TNF transgenic mice (line 3647) [28] were generously provided by Dr Edward Schwarz and permission for their use was generously provided by Dr George Kollias at the Alexander Fleming Biomedical Sciences Research Centre. TNF transgenic and C57BL/6 mice were cared for and euthanized in a manner approved by the University of Wisconsin Animal Care and Use Committee.

### Synovial fibroblast cultures

Synovial fibroblast cultures were prepared from mouse ankle joints using standard methods and from human synovial fluid according to published protocols [29]. Briefly, mice were euthanized and ankle joints were removed, minced, and incubated for 2 to 3 hours in a collagenase solution at 37°C to release cells for plating in a media of DMEM with 10% fetal bovine serum (FBS), L-glutamine, nonessential amino acids, essential amino acids, penicillin, streptomycin, and β-mercaptoethanol. Human synovial fluid was centrifuged for 10 to 20 minutes and the cell pellet resuspended in the above media. Sometimes trypsin was used to help disrupt cell clumps in synovial fluid. All synovial fibroblasts were used between passages 5 and 9. Before use, mouse synovial fibroblasts were confirmed to express vascular cell adhesion molecule-1 and not F4/80 or CD45 and human synovial fibroblasts were confirmed to express CD90 and not CD68 (data not shown). Cells were serum starved overnight before all experiments in media identical to above, but with only 0.1% FBS.

### Citrullination *in vitro*

Fibronectin was purified from human plasma by affinity chromatography as described previously [30]. This fibronectin was citrullinated as previously described by incubating 500 mg overnight at 37°C in a buffer of 100 mM Tris, pH 7.4, and 5 mM CaCl_2 _with 5 units PAD isolated from rabbit skeletal muscle (Sigma-Aldrich St. Louis, MO, USA) in a total of 1 ml [27]. As controls, fibronectin was incubated in buffer alone or in buffer with PAD that had been heat-inactivated at 95°C for 20 minutes. Heat-inactivation of citrullinating capability was confirmed by a colorimetric assay using published protocols [31]. Fibronectin samples were subjected to SDS-PAGE followed by staining with Coomassie Blue using standard methods to evaluate mobility shift.

### Fibronectin coating

Nontissue culture-treated 35 mm petri dishes (BD Biosciences San Jose, CA USA) were coated by incubation at 37°C for 1.5 hours with 10 μg/ml fibronectin, citrullinated fibronectin, or fibronectin that had been incubated with heat-inactivated PAD. Dishes were washed with PBS, blocked with 2% BSA, and washed again with PBS. Equivalent coating was assessed by coating and washing plastic dishes, and then incubating with BCA reagent (Thermo Scientific Rockford, IL USA) according to the manufacturer's instructions using a protein standard.

### Cell spreading and attachment assay

Synovial fibroblasts were incubated on plates coated with BSA, fibronectin, citrullinated fibronectin, or fibronectin exposed to heat-inactivated PAD. A 10× field that contained an average of 30 cells was chosen and time-lapse microscopy was used to image the field every minute. Every 5 minutes the percentage of cells that were no longer floating and had begun to spread and attach was assessed. Cells that were touching other cells or were not completely in the field of view were excluded.

### Migration/haptotaxis assay

Murine synovial fibroblasts (2 × 10^4^) in 0.1% FBS media were placed in the upper chamber of a transwell. The lower chamber contained 0.1% FBS media with no additive, 10 μg/ml fibronectin, 10 μg/ml citrullinated fibronectin, or 10 μg/ml fibronectin that had been incubated with heat-inactivated PAD. After 6 hours, nonmigrated cells were removed with a cotton swab and the membranes were fixed in methanol and stained with H & E.

### Western blots

For western blot of fibronectin, the fibronectin samples were run by SDS-PAGE under nonreducing conditions, transferred to nitrocellulose and blotted with the anti-fibronectin antibodies 4D1 and 5C3 [32]. For western blots of cell lysates, adherent cells were scraped into lysis buffer (5 mM Tris, pH 7.4; 500 mM NaCl; 0.1% SDS; 0.5% sodium deoxycholate; 0.5 mM MgCl_2_) on ice containing 2 μg/ml aprotinin, 1 μg/ml leupeptin, 1 μg/ml pepstatin, 0.2 mM phenylmethylsulfonyl fluoride and 1 mM sodium vanadate. For the BSA control, floating cells were used because the cells do not adhere. Equivalent amounts of cell lysates were subjected to SDS-PAGE and transferred to nitrocellulose for western blotting using anti-focal adhesion kinase (anti-FAK, clone 77; BD Biosciences), anti-paxillin (clone 349; BD Biosciences), anti-phospho-Tyr-397 FAK (polyclonal; Life Technologies, Grand Island, NY, USA), and anti-phospho-Tyr-118 paxillin (polyclonal; Life Technologies) followed by imaging with an Odyssey infrared imaging system (LI-COR Biosciences, Lincoln, Nebraska USA).

### Gelatin degradation assay

Gelatin coverslips were prepared as published previously [33]. Briefly, coverslips were treated with 50 μg/ml poly-L-lysine, then 0.5% glutaraldehyde, then 0.2% Oregon green gelatin, and then 5 mg/ml NaBH_4 _with PBS washing steps between each treatment. Coverslips were then coated with 10 μg/ml fibronectin or citrullinated fibronectin as described above. About 10,000 synovial fibroblasts from TNF transgenic mice were plated for 4 hours on the coverslips before fixation with 3% formaldehyde, quenching with glycine, permeabilization with 0.2% Triton-X 100, blocking with goat serum and incubating with mouse anti-cortactin (clone 4F11; Millipore Billerica, MA USA) followed by washing and incubating with goat anti-mouse rhodamine red. Coverslips were mounted and viewed at 60× magnification.

### Data analysis

Prism software was used for statistical analysis (Orange County, CA USA). Data were analyzed by one-way analysis of variance or unpaired *t *test as appropriate.

## Results

To assess the effect of citrullinated fibronectin on synovial fibroblasts, we first prepared citrullinated fibronectin. We used the same method of *in vitro *citrullination as Chang and colleagues, who evaluated the binding of citrullinated fibronectin to β_1 _integrin [27]. The citrullinated fibrinogen that caused increased macrophage activation, immunogenicity, and arthritogenicity was prepared using similar methods and reagents [10,12]. In brief, fibronectin was incubated with buffer containing PAD or, as a control, buffer alone. As an additional control, we used fibronectin exposed to PAD that was heat-inactivated at 95°C. Heat-inactivated PAD does not have any detectable citrullinating activity and appears to stay in solution (data not shown).

**Figure 1:**
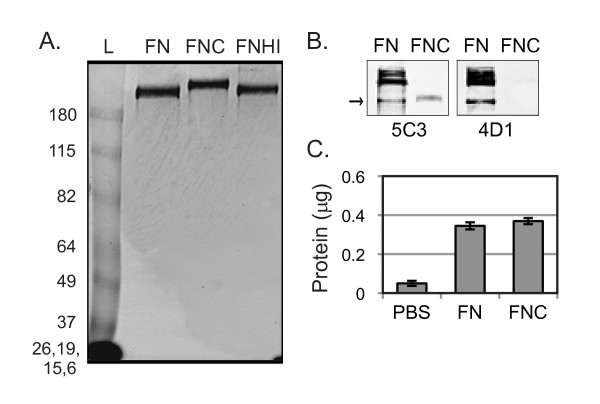
***In vitro *citrullinated fibronectin has delayed migration by SDS-PAGE, altered antigenicity, but coats plastic normally**. **(A) **Fibronectin incubated in buffer (FN), fibronectin incubated in buffer with peptidyl arginine deiminase (FNC), and fibronectin incubated in buffer with heat-inactivated peptidyl arginine deiminase (FNHI) were subjected to SDS-PAGE under reducing conditions followed by Coomassie Blue staining. Gel is representative of three experiments. Thick band at the bottom left of the gel shows the four indicated size markers combined in this 6% acrylamide gel. L, protein standard ladder. **(B) **FN and FNC were subjected to SDS-PAGE under nonreducing conditions followed by western blotting with the anti-fibronectin antibodies 5C3 or 4D1 (4D1 requires Arg107 for binding.). Blots are representative of three experiments. **(C) **Plastic wells were incubated with PBS, FN, or FNC followed by washing with PBS. Adhered protein was quantified using BCA reagent. Data shown are the average and standard error of 10 replicates from two independent experiments.

Since the anti-modified citrulline antibody used by numerous other groups has been unavailable for years, we took a different approach to assess citrullination. Citrullinated fibrinogen has been shown to have delayed migration by SDS-PAGE [10], so we assessed citrullination of fibronectin by mobility shift. As shown in Figure 1A, citrullinated fibronectin migrates more slowly by SDS-PAGE. We also confirmed the loss of arginine number 107 by western blot. To do so, we used two different antibodies against fibronectin; 4D1 recognizes an epitope in which arginine 107 is critical, and 5C3 recognizes an epitope in which no arginine has been shown to be critical [32]. Both antibodies recognize conformationally determined epitopes that are lost upon reduction [32], requiring us to run our gel under nonreducing conditions. As shown in Figure 1B, both 5C3 and 4D1 recognize the untreated fibronectin sample, but only 5C3 binds to the citrullinated fibronectin. The inability of 4D1 to bind to the citrullinated fibronectin suggests that there has been a loss of arginine at position 107. Of note, multiple bands of untreated fibronectin are detected by western blot, but only one citrullinated fibronectin band is seen. Fibronectin self-aggregates in solution [34,35], and thus the multiple bands in the untreated fibronectin lane probably represent monomers and different size multimers. Only a single monomer band of citrullinated fibronectin is detected, probably because the loss of charge via citrullination alters the ability of fibronectin molecules to aggregate. As additional evidence of citrullination, we found that citrullinated fibronectin was less degraded when treated with trypsin, which cleaves at lysine and arginine, the latter of which is converted to citrulline by PAD (data not shown).

Since citrullinated fibronectin has a reduced charge, we tested whether it coated plastic equally to untreated fibronectin. Plastic wells were incubated with PBS, fibronectin, or citrullinated fibronectin and washed with PBS. The amount of coated protein on the plastic was quantified and found to be equal for untreated and citrullinated fibronectin (Figure 1C).

**Figure 2:**
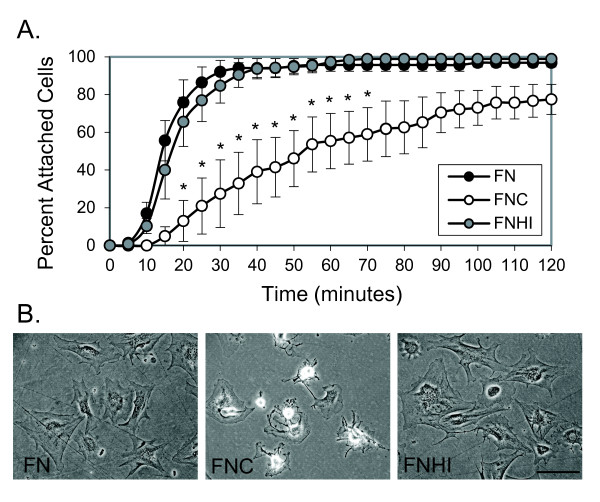
**Murine synovial fibroblasts have impaired attachment and spreading on citrullinated fibronectin**. **(A) **Murine synovial fibroblasts were plated on dishes coated with fibronectin (FN), citrullinated fibronectin (FNC), or fibronectin incubated with heat-inactivated peptidyl arginine deiminase (FNHI) and were imaged using time-lapse microscopy at 10× magnification. The percentage of cells attached in a 10× field was determined every 5 minutes. Graph depicts average and standard error at each time point (*n *= 4 experiments). *P < 0.05. **(B) **Representative images from (A) taken at 2 hours at 20× magnification. Bar = 100 μm.

To assess the ability of synovial fibroblasts to attach to citrullinated fibronectin, murine synovial fibroblasts were plated on plastic dishes coated with fibronectin, citrullinated fibronectin, or fibronectin incubated with heat-inactivated PAD. A 10× field was imaged using time-lapse microscopy. The percentage of cells that were no longer floating and had attached to the plate in the 10× field was quantified every 5 minutes. As shown in Figure 2A, murine synovial fibroblasts had delayed attachment on citrullinated fibronectin compared with control fibronectin and fibronectin exposed to heat-inactivated PAD. Furthermore, cells were less spread on citrullinated fibronectin at the end of the 2-hour experiment (Figure 2B).

**Figure 3:**
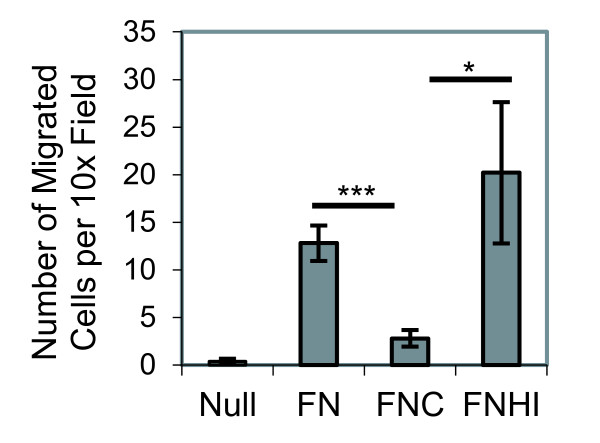
**Murine synovial fibroblasts have impaired migration towards citrullinated fibronectin**. Synovial fibroblasts were allowed to migrate for 6 hours towards no additive (Null), fibronectin (FN), citrullinated fibronectin (FNC), or fibronectin incubated with heat-inactivated peptidyl arginine deiminase (FNHI). Graph shows the average number of migrated cells per 10× field with standard error for five experiments. Ten 10× fields were counted for each sample. *P < 0.05, ***P < 0.005.

Fibronectin also mediates cell migration. To determine whether citrullination of fibronectin modulates synovial fibroblast migration, haptotactic transwell assays were performed toward a gradient of fibronectin on the lower surface of the membrane. The cells were allowed to migrate for 6 hours toward media containing fibronectin, citrullinated fibronectin, or fibronectin that had been incubated with heat-inactivated PAD. Because serum has fibronectin, which coats the transwells during the course of the experiment, it cannot be used as an attractant. As shown in Figure 3, synovial fibroblasts have impaired migration toward citrullinated fibronectin compared with untreated fibronectin or fibronectin treated with heat-inactivated PAD. We also assessed random migration of synovial fibroblasts plated on citrullinated fibronectin using time-lapse microscopy. While there is a trend towards reduced migration on citrullinated fibronectin compared with untreated fibronectin, the cells in both treatment groups randomly migrate very little and the decrease is not statistically significant (data not shown). Taken together, these findings suggest that citrullination of fibronectin impairs both synovial fibroblast attachment and migration.

**Figure 4:**
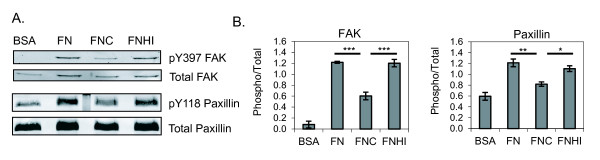
**Murine synovial fibroblasts have impaired integrin-mediated signaling on citrullinated fibronectin**. Synovial fibroblasts were allowed to adhere for 3 hours on plates coated with BSA as a negative control, fibronectin (FN), citrullinated fibronectin (FNC), or fibronectin incubated with heat-inactivated peptidyl arginine deiminase (FNHI). Cell lysates were subjected to SDS-PAGE followed by western blotting for total focal adhesion kinase (FAK), FAK phosphorylated at Y397, total paxillin, and paxillin phosphorylated at Y118. **(A) **Blots shown are representative of three experiments. **(B) **Western blots were quantified by densitometry. The amount of phosphorylated protein was normalized to total protein and this value was normalized to the overall intensity of the blots. The average and standard error are shown for three experiments. *P < 0.05, **P < 0.01, ***P < 0.005.

Fibronectin signals through integrins to activate a cascade of proteins leading to cytoskeletal changes necessary for cell adhesion and migration. Two proteins that function early in integrin-mediated signaling and become phosphorylated by integrin ligation are FAK and paxillin. Given our observations of poor attachment and migration on citrullinated fibronectin, and the fact that β_1 _integrin has decreased affinity for citrullinated fibronectin [27], we wanted to assess phosphorylation of FAK and paxillin in response to citrullinated fibronectin. We plated synovial fibroblasts on dishes coated with BSA, fibronectin, citrullinated fibronectin, or fibronectin that had been incubated with heat-inactivated PAD. Cell lysates were obtained after 3 hours and were subjected to western blotting for total FAK, phospho-FAK, total paxillin, and phospho-paxillin. Blots were analyzed by densitometry and the amount of phosphorylated protein was normalized to the amount of total protein. As shown in Figure 4, synovial fibroblasts have reduced phosphorylation of both FAK and paxillin on citrullinated fibronectin, suggesting that citrullination of fibronectin impairs integrin-mediated signaling.

**Figure 5:**
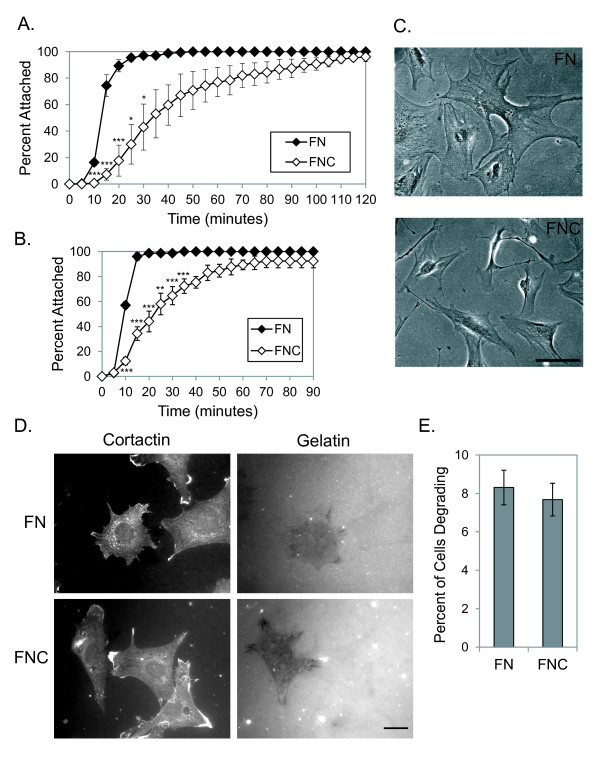
**Arthritic synovial fibroblasts have impaired adhesion and spreading on citrullinated fibronectin, but normal gelatin degradation**. **(A) **Synovial fibroblasts derived from arthritic mice were plated on fibronectin (FN) or citrullinated fibronectin (FNC) and were imaged using time-lapse microscopy at 10× magnification. The percentage of cells attached in a 10× field was determined every 5 minutes. Graph depicts average and standard error at each time point (*n *= 3 experiments). **(B) **Synovial fibroblasts derived from the synovial fluid of patients with rheumatoid arthritis were plated on FN or FNC and imaged using time-lapse microscopy as in (A). Graph depicts average and standard error at each time point (*n *= 3 experiments). **(C) **Representative images of rheumatoid synovial fibroblasts plated on FN or FNC for 3 hours. Magnification is 20×. Bar = 100 μm. **(D) **Synovial fibroblasts from TNFα-overexpressing mice were plated on coverslips coated with fluorescent gelatin and either FN or FNC. After 4 hours, coverslips were fixed and stained for cortactin (a marker of invadopodia). Representative images of degrading cells are shown at 60× with the same field shown for both cortactin and gelatin. Bar = 20 μm. **(E) **Graph depicts the percentage of cells that showed areas of gelatin degradation. For each experiment, 100 cells were evaluated per sample (*n *= 3 experiments). *P < 0.05, **P < 0.01, ***P < 0.005.

We next wanted to determine whether arthritic synovial fibroblasts also had attachment and spreading defects on citrullinated fibronectin. Therefore we assessed attachment of synovial fibroblasts from mice with chronic arthritis due to overexpression of TNFα [28]. Using identical techniques as with the normal murine synovial fibroblasts, we found that murine arthritic synovial fibroblasts have a similar delay in attachment on citrullinated fibronectin compared with untreated fibronectin (Figure 5A). Spreading was also impaired on citrullinated fibronectin (data not shown).

Even more physiologically relevant would be an abnormality in adhesion seen with rheumatoid synovial fibroblasts. We therefore isolated synovial fibroblasts from patients with rheumatoid arthritis and repeated our attachment studies. As shown in Figure 5B, adhesion was delayed on citrullinated versus untreated fibronectin for rheumatoid synovial fibroblasts. We also evaluated cell spreading 3 hours after plating cells on fibronectin and citrullinated fibronectin. Rheumatoid synovial fibroblasts had impaired cell spreading and the cells displayed an abnormal morphology with extension of spindly processes on citrullinated fibronectin (Figure 5C). For both murine and human arthritic synovial fibroblasts, results on untreated fibronectin were similar to fibronectin that had been incubated with heat-inactivated PAD (data not shown).

One of the most harmful characteristics of synovial fibroblasts is their invasive capability. Cellular invasion requires cell adhesion, migration, and the formation of invasive structures that focally degrade matrix. We were able to show defects in adhesion and migration on citrullinated fibronectin (Figures 2, 3, and 5). We therefore wanted to assess whether citrullinated fibronectin also altered focal matrix degradation by arthritic synovial fibroblasts. To this end, we plated arthritic synovial fibroblasts from mice that overexpress TNFα on coverslips that had first been coated with fluorescent gelatin and then with either fibronectin or citrullinated fibronectin. After 4 hours, cells were fixed, permeabilized, and stained for cortactin, a marker of invadopodia. As shown in Figure 5D, some cells are able to focally degrade gelatin on either citrullinated or untreated fibronectin, although they tend to move over the course of the experiment and do not align perfectly with their areas of degradation. The percentage of cells that degraded matrix was calculated and was the same for citrullinated versus untreated fibronectin (Figure 5E). Similar results were seen for rheumatoid synovial fibroblasts but a much larger percentage of cells were degrading gelatin on either substrate, making it impossible to determine whether citrullinated fibronectin increased degradation. Of note, each degrading cell had a somewhat different pattern and density of degradation, but there were no differences in the degradation patterns on control versus citrullinated fibronectin.

## Discussion

In this article we provide the first report of how synovial fibroblasts behave on citrullinated matrix, specifically how they adhere, migrate, and invade in the presence of citrullinated fibronectin. Understanding the interactions between synovial fibroblasts and citrullinated fibronectin is important for understanding the pathophysiology of rheumatoid arthritis. Fibronectin mediates many synovial fibroblast functions and is citrullinated in rheumatoid arthritis. Moreover, synovial fibroblasts are part of the destructive process of rheumatoid arthritis. However, until this work, the role of citrullinated fibronectin in synovial fibroblast behavior was unknown.

We have shown that synovial fibroblasts have impaired adhesion (Figures 2 and 5) and migration (Figure 3) on citrullinated fibronectin. Previous work has shown that fibronectin enhances adhesion of synovial fibroblasts to the rheumatoid joint, potentially leading to joint destruction. Further, migration of synovial fibroblasts in the bloodstream may contribute to the spread of rheumatoid arthritis. Given this information, one could hypothesize that citrullination of fibronectin, as is seen in rheumatoid arthritis, might be a mechanism to prevent synovial fibroblast adhesion and joint destruction as well as to inhibit migration of synovial fibroblasts and arthritis spread. Since citrullination occurs in joints affected by other forms of inflammatory arthritis, it could be a general mechanism to limit joint destruction in the setting of inflammation. In this scenario, citrullination has protective functions, which would be a novel role for citrullination since, to date, most studies of proteins citrullinated in rheumatoid arthritis have suggested a harmful role. However, the alternative might also be true with poor adhesion of synovial fibroblasts leading to more metastasis of these cells and the spread of arthritis.

Directly investigating the role of citrullinated fibronectin *in vivo *is difficult since overexpressing or knocking out PAD2 and/or PAD4 would alter citrullination of many proteins and would not address fibronectin alone. However, such studies are warranted to address the role of citrullination in general since citrullination occurs in rheumatoid arthritis and since ACPAs are pathologic. Further, chemicals such as Cl-amidine are being studied for treating arthritis and it will be important to understand all the potential effects of this chemical and similar others. Finally, citrullination occurs in multiple inflammatory conditions [8,9], in the lungs of smokers [36], and in different cancers [37], and there may be a common role for citrullination in all of these processes that is important to understand. This paper is one of the first steps in understanding the role of citrullination since fibronectin plays a critical role in the behavior of cells in inflammation and cancer.

We assessed whether citrullinated fibronectin would alter matrix degradation, a pathologic function of synovial fibroblasts in rheumatoid arthritis. We found no difference in the number of degrading cells in the presence of citrullinated versus untreated fibronectin. This was surprising to us. We had predicted either an increase in the number of degrading cells given the increased arthritogenicity of citrullinated collagen and fibrinogen, or a decrease in degrading cells due to the importance of fibronectin in matrix metalloprotease production and invadopodia stability. We performed additional experiments that showed no alteration in invasion through Matrigel invasion chambers incubated with PAD compared with untreated chambers (data not shown), but in those experiments multiple matrix proteins were probably citrullinated and the specific role of citrullinated fibronectin could not be addressed. However, those results are in agreement with the findings of our focal degradation experiments on fibronectin. Citrullination of fibronectin therefore appears to affect some synovial fibroblast behaviors but not all.

We also addressed the mechanism of impaired synovial fibroblast adhesion and migration on citrullinated fibronectin and found reduced phosphorylation of FAK and paxillin. This observation, in combination with the known decreased affinity of citrullinated fibronectin for β_1 _integrin [27], suggests that the impaired adhesion and migration is due to decreased binding of citrullinated fibronectin to integrins and subsequent decreased integrin-mediated signaling.

We tried to assess whether the canonical cell-binding RGD domain was involved with poor adhesion on citrullinated fibronectin. In competition experiments using RGD peptide to interfere with synovial fibroblast adhesion to fibronectin, however, we found that even high concentrations of RGD peptide did not significantly alter synovial fibroblast attachment in our assay. We were therefore unable to show lack of competition using a peptide with arginine replaced by citrulline (data not shown). Another domain known to mediate adhesion is the heparan sulfate binding domain. The major heparin binding sequences contain arginines [38], so this domain may be important for synovial fibroblast adhesion to fibronectin and impaired adhesion to citrullinated fibronectin. A general loss of tertiary structure with citrullination, as has been shown for other proteins [39], may also impair fibronectin's function and protein interactions and thus be responsible for altered synovial fibroblast behavior on citrullinated fibronectin. Altered tertiary structure or loss of arginines in portions of fibronectin not yet recognized to be involved with synovial fibroblast adhesion may be the most probable explanation for the observed cellular defects on citrullinated fibronectin since, to date, only five arginines in human fibronectin purified from the synovial fluid of two people with rheumatoid arthritis have been identified as citrullinated [40], none of which are in the RGD domain or the heparan sulfate binding domain.

One important caveat in interpreting our studies, however, is that the effects of *in vitro *citrullinated human plasma fibronectin by PAD from rabbit skeletal muscle on mouse and human synovial fibroblasts might be different from the effects of physiologic citrullination of fibronectin on synovial fibroblasts in the inflamed human joint. Nonetheless, the alterations of synovial fibroblast behavior on citrullinated fibronectin that we report here open the doors to future studies.

## Conclusion

This is the first paper to show how citrullinated fibronectin, which is present in rheumatoid arthritis, alters the behavior of both normal and rheumatoid synovial fibroblasts. Given the pathologic role of ACPAs in rheumatoid arthritis, the presence of citrullination in inflammation and cancer, and the importance of communication between cells and extracellular matrix proteins, the implications of this work may be profound. However, further studies are needed to address the role of citrullinated fibronectin and other citrullinated extracellular matrix proteins in arthritis and other diseases both *in vitro *and *in vivo*.

## Abbreviations

ACPA: anti-citrullinated protein antibody; BSA: bovine serum albumin; CXCL: chemokine (C-X-C motif) ligand; DMEM: Dulbecco's modified Eagle's medium; FAK: focal adhesion kinase; FBS: fetal bovine serum; H & E: hematoxylin and eosin; IL: interleukin; PAD: peptidyl arginine deiminase; PBS: phosphate-buffered saline; TNF: tumor necrosis factor.

## Competing interests

The authors declare that they have no competing interests.

## Authors' contributions

MAS conceived of the study, designed all of the experiments, participated in the acquisition of all data, analyzed and interpreted all data, and wrote the manuscript. DAB helped to design western blot experiments, helped to acquire western blot data, helped to analyze western blot experiments, and drafted part of the manuscript. DFM contributed to experimental design, data interpretation and maturation of the manuscript. AH contributed to experimental design, data interpretation and revised the manuscript. All authors read and approved the final manuscript.
